# Roles of Mechanosensitive Channel Piezo1 in Wound Healing and Scar Formation

**DOI:** 10.3390/life14030377

**Published:** 2024-03-13

**Authors:** Hans-Oliver Rennekampff, Mayer Tenenhaus, Isabelle Rennekampff, Ziyad Alharbi

**Affiliations:** 1 Department of Plastic Surgery, Hand and Burn Surgery, Rhein Maas Klinikum, 52146 Wuerselen, Germany; 2 Independent Researcher, San Diego, CA 92107, USA; 3 Dental School, RWTH Aachen, 52062 Aachen, Germany; isabelle.rennekampff@rwth-aachen.de; 4 Plastic Surgery and Burn Unit, Fakeeh College for Medical Sciences, Dr. Soliman Fakeeh Hospital, Jeddah 23323, Saudi Arabia; zialharbi@fakeeh.care

**Keywords:** Piezo1, ion channel, wound healing, scar, mechanobiology

## Abstract

The ability to heal one’s wounds is perhaps one of the most fundamental and critical of physiologic processes. This coordinated and closely regulated sequential biological process involves a variety of migratory and resident cells. The activation, modulation, balance, and control of these functions depend upon soluble mediators that activate cells and modulate their diverse functions. Recent advances have identified mechanotransduction as functionally integral in many different cell types and physiologic processes. The mechanically sensitive ion channel Pieoz1 is expressed on platelets, neutrophils, macrophages, endothelial cells, keratinocytes, and fibroblasts, all of which are principally involved in wound healing. On a cellular level, there have been great advances in our understanding of the functional role of Piezo1 mechanotransduction in cutaneous wounding. The blocking of Piezo1 has recently been shown to reduce scarring in vivo and yet, thus far, a comprehensive understanding of the roles that Piezo1 plays in in vivo wound healing remains lacking. Recognizing the ever-present and critical importance of optimal and reparative wound healing, and with the availability of new physical mechanomodulating devices, the time is ripe for gaining deeper insights into optimizing wound healing. In this review, we describe the current knowledge of Piezo1 related to wound healing.

## 1. Introduction

Upon injury to the skin, a well-orchestrated sequence of events is set in motion to repair and close a wound [1,2,3]. The mechanisms underlying wound healing involve a number of resident and migratory cell types such as keratinocytes, fibroblasts, neutrophils, and macrophages. Secreted soluble mediators such as growth factors and cytokines modulate and control their actions, while matrix components such as fibronectin and collagen re-establish integrity. Biochemical factors and biophysical cues, such as electrical wound currents [4,5] and mechanical forces [6,7,8], are increasingly being recognized as playing an important role in all phases of wound healing.

The cutaneous healing process is classically described as executed in three synchronized and overlapping phases—the inflammatory phase, proliferative phase, and maturational phase—which ultimately terminate in a restorative remodeling phase if all goes well. The initial inflammatory phase can be divided into four major events: coagulation, fibrinolysis, the extravasation of polymorphonuclear neutrophils (PMNs), and macrophage recruitment. Thrombus formation at the site of injury, facilitated by platelet adhesion, leads to additional platelet aggregation and the secretion of a number of wound-modulating factors [9]. Following thrombus formation, PMNs and some monocytes migrate to the wound. PMNs, the most abundant circulating blood leukocytes, function as effectors of inflammation and provide a first-line of defense against infection [9,10]. Macrophages replace PMNs in the wound and are primarily responsible for the phagocytosis of pathogenic material and necrotic debris, matrix degradation, as well as remodeling. Their critical importance in wound healing and their central role in regulating the ensuing proliferative phase is affected by factors that stimulate angiogenesis and fibroplasia [1,2,9,11,12,13]. As wounds heal, the local macrophage population transitions from a predominantly pro-inflammatory (M1-like phenotypes) cell type to that of an anti-inflammatory (M2-like phenotypes) cell type [1,11,14]. The proliferative phase characterized by angiogenesis, fibroplasia, and re-epithelization follows, depending on the interrelated cell types, expressions, processes mediated by the local environment (pH, oxygen tension, cytokines) and external determinants. Angiogenesis describes the vascularization of the previously avascular provisional wound matrix. This requires directed cellular migration, proliferation, and capillary formation [15], which is stimulated by the relative hypoxic state, promoted by a variety of chemotactic factors and facilitated in remodeling by associated proteases. A few days into the healing process, fibroblasts begin to migrate from the wound edge and underlying tissue into the wound site where they populate and proliferate, initiating the proliferative phase of repair. A subset of these cell types, myofibroblasts, adopt contractile properties (alpha-smooth muscle actin), rendering them responsive to extracellular cues. Abundantly found in granulation tissue and responsible for wound contraction [2] these cells are implicated in the formation of scars [16]. The epithelialization of cutaneous wounds is a coordinated process of keratinocyte migration and proliferation, as well as final differentiation [1,3]. Epithelial migration generally begins from the wound margin and proceeds inwards, augmented by adnexal contributions.

Disturbances or the discoordination of any of the phases of wound healing may lead to untoward wound healing sequalae, such as hypertrophic scarring and chronic ulcers. Understanding the cues that modulate and regulate wound healing is of particular clinical importance if we are to develop improved preventive measures and therapeutic options. Recombinant growth factors have been extensively studied and promoted in an effort to modulate and optimize wound healing with the hope of preventing or at least minimizing pathologic scarring [17,18] and ulcerations. However, thus far, and despite promising study results, these therapeutic approaches have not shown significant benefits or resulted in widespread clinical adoption. 

Recently, biophysical modalities, such as shock wave therapies [19,20] and mechanical devices [21], have received particular attention for modulating wound healing. Numerous lines of evidence relate mechanical force as sensed by fibroblasts to downstream events such as excessive scarring [22,23,24,25,26]. Several membrane-bound mechanosensory receptors such as integrins have since been described [6,27] whereby mechanical forces are transmitted across the cell membrane activating downstream events such as cell motility, cellular proliferation and protein synthesis.

**Figure 1:**
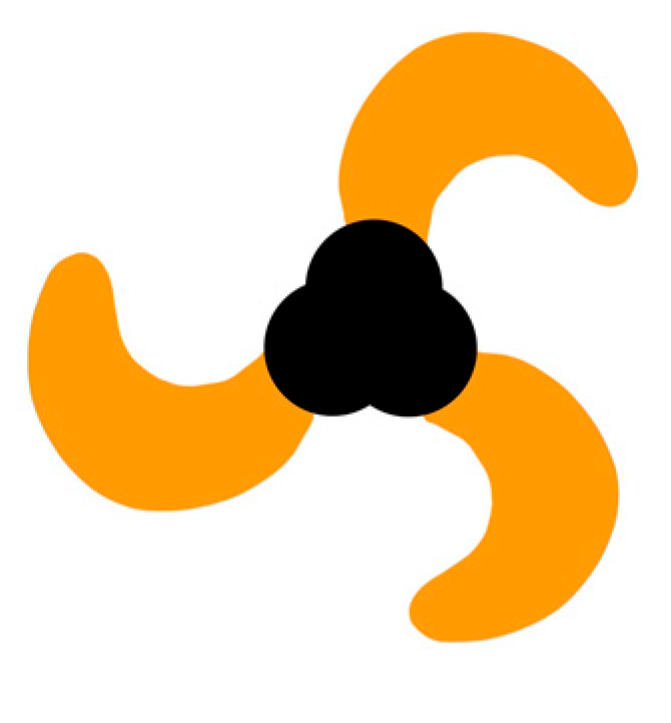
Schematic illustration of Piezo1 as visualized from the top (extracellular) view. The three C-terminal extracellular domains that form the cap (black) and the three-bladed propeller subunits (orange) are depicted (adapted from [35]).

**Figure 2:**
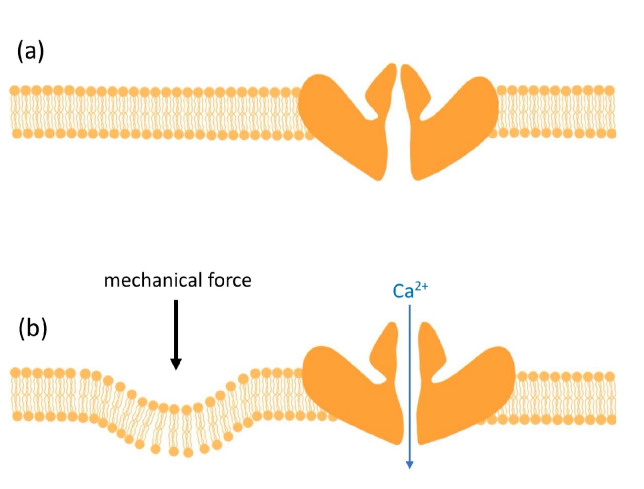
Mechanotransduction (by various modes; see Figure 3) resulting in opening of the pore with resultant calcium flux: (**a**) closed Piezo1 channel; (**b**) open Piezo1 channel (adapted from [36]).

The Nobel prize winning discovery of Piezo1 by Ardem Patapoutian has had a substantial impact on our understanding of clinical wound healing and led to a great number of highly cited publications [28,29]. Piezo1 (Fam38A) and the similar Piezo2 (Fam38B) are cation-selective ion channels with rapid voltage-dependent inactivation [30,31]. The murine Piezo1 consist of two modules: a peripheral mechanotransduction portion that resembles a three-bladed propeller and a central pore formed by three C-terminal extracellular domain caps responsible for ion flux [32] (Figure 1). Initially identified in a neuroblastoma cell line [30], these primary mediators of mechanotransduction are expressed in many organs, such as the lungs, gastrointestinal system and vasculature, as well as many different cell types [30,31] such as keratinocytes, fibroblasts, and endothelial cells, the latter of which are all involved in wound healing. Of late, a large body of research has expanded our understanding of their involvement in processes such as skin homeostasis and proliferation, with particular focus on tumor regulation, malignant spread and immunity [33,34]. Mechanical activation of Piezo1 from vectored forces such as shear stress, swelling, tension, and compression have been shown to result in pore opening and calcium-permeable cationic currents, promoting downstream cellular signals (Figure 2). This transduction of force can occur through cytoskeletal tethers (also known as force-through-filaments) or through the membrane (also known as force-through-lipid). It is interesting to note that Piezo1, while transmembranously expressed, can also be found in the endoplasmic reticulum, mitochondria, and nucleus, suggesting a particularly complex inter-relationship [33].

In this comprehensive review, we describe findings on the mechanisms of mechano-sensation (i.e., signal detection), mechano-transduction (i.e., signal transmission) and downstream biological responses by the Piezo1 channel as they relate to cutaneous wound healing.

## 2. Inflammation Phase

### 2.1. Platelets

Elevated shear stress on platelets has been shown to significantly contribute to thrombus formation and its subsequent activation [37]. In pathologic responses, this activated state may manifest in Virchow’s triad characterized by intravascular vessel wall damage, stasis, and a hypercoagulable state. On a cellular level, shear stress on platelets has been shown to result in a 10-fold increase in intracellular calcium [38,39], which accumulates in the developing thrombus and is accompanied by platelet aggregation. When platelets sense microenvironmental mechanical properties, such as substrate stiffness, this interaction results in transduced differential biological signals and further activation [40]. 

Ilkan et al. [38] described the expression of the Piezo1 ion channel in platelets at both the mRNA and protein level and demonstrated the functionality of this channel ion by shear-induced calcium flux and Piezo1-dependent thrombus formation. Conclusively, Yoda1, an agonist of Piezo1, stimulated calcium entry into platelets, whereas GsMTx-4, a Piezo1 antagonist, inhibited ion flux. Elevated Piezo1 expression on platelets was reported to exert a prothrombotic effect [41]. In vivo, Zhao et al. [39] demonstrated that the systemic application of the antagonist GsMTx-4 to Piezo1 resulted in an antithrombotic effect in mice. Furthermore, the authors reported that Piezo1 activation affected platelet mitochondrial function and was also involved in platelet αIIbβ3 integrin activation. This outside-in signaling by αIIbβ3 initiates multiple cellular events, such as spreading, thrombus consolidation, and clot retraction [42]. 

### 2.2. Polymorphonuclear Neutrophils

The functional and mechanical relationship of polymorphonuclear neutrophils (PMNs) and vasculature has long been well documented and thought to represent a prerequisite for PMN function [43,44,45]. Several inflammatory and anti-inflammatory mediators such as interleukins are now well recognized for their effects as both modulators and activators of PMNs. Chase et al. [46] demonstrated that the IL-8 induced arrest of PMNs rolling on E-selectin, a selectin cell adhesion molecule expressed on endothelial cells and is associated with a rapid increase in intracellular calcium. This strong interplay between biochemical stimulation, adhesion receptors, and the mechanostimulation of PMNs is further underlined by findings that a shear stress of 2 dynes/cm^2^ increased IL-8 induced calcium flux, while PMNs in the normal circulation are only minimally activated or even deactivated [46,47,48]. When PMNs transmigrated through the endothelial cell layers, an increase in calcium influx was noted [49]. In accordance with these complex interrelationships, the importance of calcium channels has gained particular prominence [50,51]. 

Piezo1 expression was detected in the PMNs of mice and humans [41,49], with higher levels of Piezo1 expression in diabetic conditions. Zhu et al. [41] showed that PMNs can be mechanically activated via Piezo1, resulting in evoked currents, the release of neutrophil elastase, and chromatin swelling, demonstrating a direct association between Piezo1 and a mechanothrombotic pathway in diabetes, suggesting possible therapeutic strategies. Very recently, Mukhopadhyay et al. [49] demonstrated that mechanically activated PMNs show increased bactericidal activity both in vitro as well as in vivo. Whether this Piezo1-activated enhanced host-defense pathway with induced expression of Nox4 is also active in hyperglycemia-induced Piezo1 upregulation remains to be investigated.

### 2.3. Macrophages

Recently, our understanding of the contributions of macrophages to the process of wound healing has expanded greatly [11,12,13,14,52]. It is now well known that during the course of normal wound healing, the local macrophage population transitions from a predominantly pro-inflammatory M1-like phenotype to anti-inflammatory, pro-healing M2-like phenotypes [53,54]. However, it is important to recognize that these varied polarized forms, dependent on both location and state, participate, promote, and modulate both favorable and unfavorable physiologic and pathologic processes. 

During the early inflammatory phase, a depletion of macrophages generally results in suboptimal granulation tissue formation and impaired wound closure while depletion during the proliferative stage of wound healing has been shown to result in hemorrhage as well as impaired tissue maturation [11]. In clinical practice, it is well known that a paucity of M2 macrophages, as so often observed in challenging cases of diabetes mellitus, results in significantly impaired wound closure [14]. While biochemical signals have been shown to play an important role in polarization toward the macrophage phenotype, there is now increasing evidence that mechanical forces likely represent another significant factor responsible for the polarization of macrophages into the two major subtypes [55,56,57,58]. Macrophages, when cultured on stiffer matrices, shift to an activated phenotype [56,57]. Zhao et al. [58] reported that Piezo1 promoted macrophage polarization toward the M1 type in response to lipopolysaccharide (LPS) with the subsequent production of proinflammatory cytokines. Solis et al. [59] found that applying cyclical hydrostatic pressure upregulated proinflammatory genes, such as *Il1b*, *Cxcl10*, and *Ptgs2* in macrophages. Further experiments have gone on to demonstrate that this mechanosensing is Piezo1-dependent. 

Piezo1-mediated calcium signaling regulates macrophage polarization leading to activator protein-1 (AP-1) activation, the production of endothelin-1 (EDN1), and stabilization of hypoxia-inducible factor 1α (HIF1α), with subsequent production of pro-inflammatory mediators (IL-6, TNF-α, prostaglandin E2) in response to IFNγ/LPS stimulation [59,60]. In line with these findings, macrophages with depleted Piezo 1 secreted significantly less TNFα, IL6, and inducible nitric oxide synthase. Taken together, these observations help us to understand and appreciate how the environment such as that found in lung fibrosis results in a continuous proinflammatory milieu [59]. It is intriguing to speculate that the thickened and fibrotic wound bed so often seen in chronic wounds exerts a similar detrimental effect on macrophages. In clinical practice, the stiff and fibrotic sequalae observed in many patients with longstanding chronic wounds has long been recognized as resulting from a stagnant and yet protracted inflammatory process. These wounds are in fact characterized by an overwhelming proinflammatory phenotype that likely manifests in the very woody presentation so readily evident upon clinical examination. This fibrotic state and cellular makeup with ongoing mechanostimulation and subsequent proinflammatory cytokine secretion likely participate in the resultant vicious cycle typically observed in chronic wounds. 

Piezo1-induced proinflammatory cytokine secretion has been shown to induce the production of downstream matrix metalloproteinases (MMP) in fibroblasts with subsequent collagen degradation [58]. Whether MMP degradation of the (stiffer) matrix represents a relevant feedback loop is has not yet been fully examined.

Further experiments on macrophage bactericidal activity suggested that Piezo1 is also required for actin cytoskeletal reorganization and the production of reactive oxygen species (ROS), a prerequisite for phagocytic activity [61]. With the subsequent depletion of macrophages in Piezo1, a reduction in bacterial phagocytosis in vitro and in vivo occurs. 

In line with the role of Piezo1 in mechanobiology, stiff substrates have been shown to enhance the bacterial killing of macrophages in vitro. This effect was abrogated in Piezo1-deficient cells [61]. Yoda1 is the first agonist developed for Piezo1. While agonistic stimulation with Yoda1 clearly increased phagocytosis, there is evidence that the assembly of a complex of Piezo1 with additional receptors such as TLR4, one of the pattern recognition receptors responsible for activating the innate immune system, is a prerequisite for the induction of calcium influx and bactericidal activity of macrophages [61,62]. Similarly, the crosstalk of the integrin CD11b with Piezo1 seems necessary to exert proinflammatory protein expression. Atcha et al. [63] reported that the knockdown of either CD11b or Piezo1 abrogated stretch-mediated changes in inflammatory responses. 

In contradistinction to several of these observations, it is interesting to note that a number of other studies have observed that stiff substrates appear to prime macrophages toward the M2 phenotype (summarized in [64]). It has been speculated that, in addition to contributions of the matrix proper, additional interfering factors, such as culture conditions and source, likely cause these conflicting results. Of particular note is the recognition that the mode of mechanostimulation, such as pressure, stretch, or matrix properties, determine the Piezo1-dependent function of macrophages. Whereas a stiff matrix leads to an increase in inflammatory mediators, both static and cyclic stretch suppressed IFNγ/LPS-induced inflammation. In their study, Atcha et al. [63] demonstrated that mechanical stretch downregulates Piezo1 expression, thus suppressing IFNγ/LPS-mediated inflammation. Additional experiments revealed that cyclic stretch alone did not drive macrophages towards activation. 

Interstitial flow, in addition to matrix stiffness and stretch, all commonly impaired in problem wound healing states [65], have been identified as contributing factors in the modulation of the macrophage phenotype [55]. When interstitial flow (~3 μm/s) is applied to macrophages, it drives macrophages toward an M2-like phenotype (CD163, CD206 positive) with enhanced expressions of TGFβ, arginase-1, and transglutaminase 2 [55]. Arginase-1, a metalloenzyme that catalyzes the conversion of arginine to ornithine and urea in the final step of the urea cycle, is principally involved in balancing collagen and polyamine production and considered critical for wound repair and regulation [66]. 

Follow-up experiments have suggested that M2 polarization is mediated via β1 integrin/Src-dependent STAT3/6 activation [55]. STAT6, a member of the Signal Transducer and Activator of Transcription family of proteins transmit signals from a receptor complex to the nucleus and activates gene expression. While the involvement of Piezo1 was not specifically tested in these experiments, the crosstalk between β1 integrin and Piezo1 is with decreased expression of integrin β1 noted in Piezo1-deficient cells well-described [67]. Atcha et al. [60] described a similar increase in STAT6 in IL4/IL13-stimulated macrophages (M2) depleted of Piezo1, which correlated with a decrease in the nuclear factor kappa-light-chain enhancer of activated B cells (NF-kB), as seen in Piezo1-depleted macrophages after stimulation with IFN-γ/LPS. In addition, the authors reported that both inflammatory (M1) and healing (M2) pathways increased as substrate stiffness increased. They concluded that stiffness does not enhance inflammation at the expense of healing, but instead promotes overall macrophage responses to soluble cues. Notably, the activation of Piezo1 with the agonist Yoda1 inhibited the M2 macrophage response on stiff substrates suggesting a different mechanosensing channel. 

It is intriguing to speculate that blocking or at least modulating Piezo1 response might prove beneficial in clinical situations where pro-inflammatory-activated macrophages dominate and are continuously stimulated, causing detrimental effects such as the production of reactive oxygen species (ROS).

## 3. Proliferation Phase

### 3.1. Keratinocytes

In an effort to generate additional skin (as well as other tissue types) for reconstructive procedures, plastic and reconstructive surgeons have long employed various forms and devices for tissue expansion [68,69]. These techniques ultimately capitalize on concepts of mechanotransduction to promote new cell growth [68]. In tissue expansion, the physical or mechanical stretch of skin, as well as fluid compression and extrusion, are clearly evident to the observer and yet reflect only small contributions to the ultimate creation of additional tissue. The mechanical stretching of keratinocytes at the wound site has been linked to the proliferation of keratinocytes [70,71,72,73,74,75]. When observed in a monolayer keratinocyte culture, stretching results in profound morphological changes in addition to enhanced proliferation [70,72] and stretch-stimulated, cultured human skin equivalents have been shown to develop a thicker epithelium compared to non-stimulated constructs [73]. This finding correlates well with what is clinically observed when skin is subjected to tissue expansion [76].

In concert with these events, mechanotransduction in keratinocytes has been linked to a variety of membrane receptors, such as integrins, growth factor receptors, G-protein-coupled receptors, ion channels, and cell–cell adhesion molecules such as E-cadherin [77]. When, for example, β1 integrins are activated, they are shown to mediate rapid downstream ERK1/2 activation as well as YAP activation, ultimately resulting in cell proliferation [70,71]. E-cadherin has similarly been linked to mechanotransduced mitotic activity in kidney epithelial cells [78]. In their experiments, Benham-Pyle et al. [70] were able to demonstrate that stretch-activated YAP1, a transcription coregulator component in the hippo signaling pathway, was required for cell cycle re-entry, whereas β-catenin induced by cadherin degradation was necessary for progression from G1 to S phase. Yano et al. [72] reported that stretching keratinocytes led to the phosphorylation of the epidermal growth factor receptor (EGFR) with the downstream activation of the ERK1/2 (mitogen and extracellular signal-regulated kinase) and PI 3-K (phosphoinositide 3-OH kinase) pathways, with subsequent mitotic activity potentially caused by S phase entry. More importantly, the authors demonstrated that calcium influx was indispensable for the activation of ERK1/2 by mechanical stretching, intimating the importance of ion channels in stretch-induced mitotic activity. 

Building upon and supporting these observations, Gudipaty et al. in 2017 [74] demonstrated Piezo1 ion channel activation in mechanical-stretch-induced cell division. Piezo1 has been functionally explored in keratinocytes and linked to mechanotransduction in this cell type [74,79,80,81]. When challenged via antagonistic inhibition as well as the siRNA-mediated knockdown of Piezo1, the requirement of Piezo 1 in stretch-induced cell division was reinforced [74]. The authors were able to show that stretch-activated Piezo 1 in epithelial cells led to Ca^2+^ influx, which in turn led to the phosphorylation of ERK1/2, thereby activating the cyclin B mandatory for cell cycle progression at the end of G2. Collectively, these reports on different proliferative pathways and different mechanoreceptors might explain the biphasic stretch-induced proliferative response of keratinocytes with mitotic events occurring as early as 1 h post stretching, possibly by G2/M transition, and a second peak at approximately 15 h post stretching, possibly by a G1 reentry of resting cells and further progression into the S phase.

A number of different mechanisms for the migration of keratinocytes such as leapfrogging, dragging, or crawling at the wound edge have been proposed [1,3], all of which might exert mechanical force on keratinocytes at the leading front or pull on the epidermis behind the migrating epithelium. Additionally, the retraction of the wound edge, so commonly observed in full thickness wounds, likely generates yet another mechanical force on the epithelium. Consistent with these assumptions, the elongation of keratinocytes has well been described and detected early after wounding at the wound edge [82]. 

Migratory keratinocytes acquire a different phenotype, and this epithelial–mesenchymal transition in keratinocytes is characterized by cytoskeleton rearrangement, the loss of adhesive junctions, apical–basal polarity, changes in adhesion molecule expression, as well as a shift in cytokeratins [1,3,81,82,83]. The expression of the cytokeratin pair K6/K16 in keratinocytes at the wound edge reflects a highly activated stage and the stretching of keratinocytes has been shown to induce K6 expression in vitro [72]. He et al. [81] presented additional data on stretch-induced cell changes, such as the upregulation of N-cadherin, vimentin, fibronectin, and α-SMA, all of which are hallmarks of epithelial–mesenchymal transition, and were alleviated by blocking Piezo1. Furthermore, the authors found that blocking Piezo1 by Piezo1 knockdown or GsMTx4 abrogated stretch-induced keratinocyte migration. In line with these results, the force-related upregulation of Matrix metalloproteinase 2 (MMP2) and MMP9 expression was noted, both of which are critical for cell migration [81,84]. Stretch-induced MMP2 and MMP9 expression was downregulated by blocking Piezo1 [81]. In sharp contrast, Holt et al. reported that the conditional knockout of Piezo1 in keratinocytes led to an increased migratory profile in vitro. Subsequent in vivo experiments revealed an increased rate of wound closure in epidermal-specific Piezo1 knockout mice. These seemingly contradictory results might be explained by virtue of the different experimental set-ups employed (e.g., stretch vs. non-stretched). Further experiments on the role of Piezo 1 in in vivo wound healing models seem necessary. While in vitro experiments demonstrate the important role that Piezo1 plays in keratinocytes, the distribution of Piezo1 expression in human wounds during reepithelization remains to be comprehensively investigated.

### 3.2. Endothelial Cells

Endothelial cells have been shown to functionally express Piezo1 when presented with a variety of mechanical stimulation forces or types, such as fluid shear stress and elevated micro vessel pressure, leading to an influx of Ca^2+^ with a resultant further increase in the membrane density of Piezo1 channels [85,86,87,88,89]. A critical interplay with other mechanosensors, such as TRPV4 and integrins α5β1, αvβ3, and αvβ5, has been shown [90,91,92]. It is now well known that Piezo1 can act as a flow sensor in inflammation, vascular development, and adult vascular physiology [93]. The depletion of Piezo1 in human umbilical vein endothelial cells and endothelial tip cells inhibits the migration of cells, subsequent tube formation and vascular pattering, respectively [87,88], both critical processes in vessel formation and wound healing, where impaired angiogenesis can result in chronic nonhealing wounds. In line with the angiogenic effect, Piezo1 has been shown to induce the sprouting of vessels mediated by membrane type 1 matrix metalloproteinase-1 and matrix metalloproteinase-2 activation [94]. In vivo experiments by Ichioka et al. [95] demonstrated that shear stress induced an angiogenic effect. 

Numerous modalities for effecting mechanical stress have been tested in an effort to increase angiogenesis with the intent of optimizing tissue perfusion and wound healing. It has long been recognized that wall shear stress plays a pivotal role in the vascular system physiological adaptation. In the wound healing arena, this effect is used clinically to promote a favorable physiologic response by employing negative pressure [96,97]. Whether this effect is Piezo1-dependent or not has not yet been examined. 

As critically reviewed by Huang et al. [98], Piezo1 on endothelial cells appears to exert characteristic effects that can be both beneficial as well as harmful. These differential effects of Piezo1 on endothelial cell function appear to be dependent on flow and pressure kinetics [99,100,101,102,103] as well as matrix composition [92]. Accordingly, the upregulation of Piezo1 has been linked to both atherosclerosis and inflammation, whereby disturbed blood flow leads to Piezo1- and Gq/G11-mediated integrin activation, resulting in NF-κB activation and atherogenesis [101,102,103]. Interestingly, in vivo atherosclerotic plaques have been associated with Piezo1 expression and inflammatory proteins, such as NF-κB and TNF-α [104]. Similar YAP/TAZ, now also considered mechanotransducers, have been shown to be activated by disturbed flow and potentially play a critical role in blood vessel pathology [105,106]. 

The role of Piezo1 in tissue inflammation is further underscored by an evolving appreciation of PMN Piezo1-dependent diapedesis in vivo. Wang et al. [107] demonstrated that the activation of Piezo1 on endothelial cells led to the phosphorylation of tyrosine kinases sarcoma (SRC) and protein tyrosine kinase 2 (PYK2), resulting in the opening of the endothelial barrier. Recent studies point towards the role of Piezo1 in junctional remodeling between neighboring endothelial cells involving or perhaps mediated by PECAM1 (Platelet Endothelial Cell Adhesion Molecule 1 or CD31) and Cadherin-5 [108]. A similar effect on junctional stability was observed in vivo in lung capillaries after vascular pressure increase [109]. The endothelial-specific deletion of Piezo1 reduced lung capillary leakage [109]. At present, the precise role of the additional stimulation or blocking of Piezo1 in endothelial cells in wound healing remains to be established.

### 3.3. Fibroblast

Our appreciation of the relevant force-mediated aspects of Piezo1 on fibroblasts, particularly related to wound healing, has similarly been rapidly evolving. It is interesting to reflect on the fact that Piezo ion channels were extensively found on cardiac fibroblasts [93,110,111], and most recently, in a wide variety other organ systems and cell types, such as fibroblasts derived from normal human skin [112]. The range of these findings points to the incredible capacity, facility, application and potential of these entities and interactions.

As observed in functioning cardiac-derived cells, the in vitro testing of dermal fibroblast Piezo1 experienced calcium influx when undergoing mechanical stress [112]. In vitro activation by cyclic mechanical stretch has similarly demonstrated a proliferative response [112]. These effects are alluded to in numerous laboratory and clinical studies on negative pressure wound healing modalities whereby microdeformation by 125 mmHg suction resulted in fibroblast mitogenesis [113]. 

While enhanced motility was noted in stretch-activated primary human fibroblasts [112], Piezo1 activation in mouse transformed 3T3B-SV40 fibroblasts inhibited cell migration [114]. However, experiments on the force-induced differentiation of fibroblasts to myofibroblasts have demonstrated more uniform results [112,115,116,117,118]. While the Transforming Growth Factor-β1(TGF-β1) is considered to be the principle soluble factor involved in myofibroblast differentiation, there is strong evidence that mechanical force plays a major role in fibroblast-to-myofibroblast differentiation. Myofibroblasts represent a cell type which arises in the granulation phase of wound healing and exerts a force that promotes wound contraction. Reciprocally, the stiffness of the matrix has also been shown to contribute to fibroblast-to-myofibroblast differentiation [24,119]. He et al. demonstrated that cyclic mechanical loading resulted in Piezo1-dependent enhanced alpha–smooth muscle expression in fibroblasts [112]. Further studies demonstrated that cyclic mechanical stress also increased the secretion of fibronectin, collagen I, and collagen III [112], all of which were abrogated through Piezo1 knockdown. These results highlight the important role of Piezo1 in the granulation phase of wound healing, with mechanical stimuli not only increasing the number and function of cells for wound closure but also promoting the production of the matrix for enhanced migration and ultimate wound closure. 

## 4. Maturation Phase

### Fibroblasts

Extensive experimental and clinical research has described mechanical loading as a significant and contributing factor in the development of hypertrophic scarring, with an increase in both cell density and volume [6,118,120,121,122]. In experiments conducted by He et al., a link between Piezo1 and excessive matrix production by dermal fibroblasts was accurately shown [112,119]. When hypertrophic scar tissue was examined, an overexpression of Piezo1 more than threefold than that seen in normal skin was found. In their studies, the authors demonstrated that fibroblasts cultured on a stiff matrix secreted more L-proline, a requisite precursor for collagen synthesis. Additional experiments revealed that mechanical stretch was accompanied by collagen I and III production [112]. The functional involvement of Piezo1 in this viscous cycle was accurately demonstrated by experiments that blocked Piezo1. GsMTx4 and siRNA have been shown to inhibit the aforementioned mechanical-stress-induced effects. In animal experiments, GsMTx4 was able to reduce stretch-induced de novo scarring by 50% [112]. Taken together, these results help us to appreciate the important role that Piezo1 plays in scar tissue formation and possibly hypertrophic scarring. 

There is consistent evidence that hypertrophic scarring can, at least in part, be explained by evading the apoptosis of fibroblasts [123,124]. While stretch has not shown an apoptotic effect on human dermal fibroblasts [112], mechanical stress in vaginal wall fibroblasts has been shown to lead to apoptosis by disintegration of the actin cytoskeleton [125]. Fibroblasts, even in organs such as the skin, are a very heterogenous population, and as such, it is important to note that some of these effects appear to be organ-specific [126]. Furthermore, apoptotic effects are likely dependent on the type of force (stretch vs. compression), as compression has been shown to result in apoptosis of fibroblasts in vitro [127], while a similar force (15–25 mmHG) has proven effective in clinically applied scar compression (amelioration) therapy [128]. While compression as well as stretch can be sensed by Piezo1 [129], the differential role of the mechanosensor Piezo1 in the formation and resolution of scar tissue remains to be investigated. 

## 5. Clinical Perspectives

**Figure 3:**
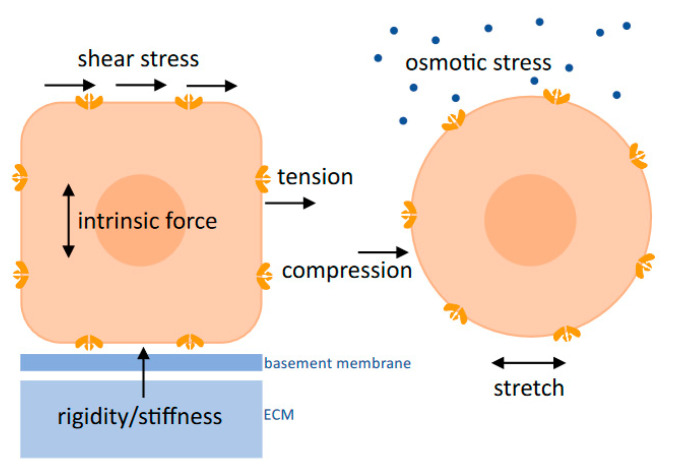
Piezo1 mechanotransduction in wound-related cells and associated force relationships, which have been described as opening the Piezo1 ion channel.

Classical teaching has long contributed to our understanding of wound healing and its temporal regulation by a number of cytokines and growth factors acting on cells, such as PMN, macrophages, endothelial cells, fibroblasts, and keratinocytes. The reflection of force vectors have led many scholars [130], including the present authors, to gaining a better understanding of physical force on wound healing. Overall, our review provides several new insights into the (patho)physiological mechanisms of mechanomodulation by the ion channel Piezo1 on platelets, PMNs, macrophages, keratinocytes, endothelial cells, and fibroblasts, all of which are involved in cutaneous healing and may well inspire new ideas for the treatment of wounds (Figure 3).

Mechanostimulation by acoustic shockwaves such as those delivered by extracorporeal shock wave therapy (ESWT) has been well described and applied as an adjunctive therapeutic measure for a variety of musculoskeletal conditions, such as bone healing in both humans and animals [131]. In both in vitro as well as in vivo settings, shock wave therapy on endothelial cells has been shown to result in improved angiogenesis [19]. It is certainly conceivable that these effects might prove beneficial when applied therapeutically as treatment adjuncts in inadequate or critical vascular perfusion settings such as those commonly observed in challenging wound types such as coronary arterial disease [132] and critical limb ischemia [133]. Extracorporeal shock wave therapy has been applied clinically for the management of burn wounds [20] and chronic wounds [134] demonstrating some promising results with enhanced epithelialization and wound closure noted, respectively. Bolstering this promise are in vitro studies which have demonstrated improved keratinocytes and fibroblasts migration and proliferation in, respectively [135]. While extracorporeal shock wave therapy represents a promising clinical therapeutic adjunct, experimental evidence for the involvement of Piezo1 is lacking. 

Elucidating the clinical potential of mechanosensors and low-intensity pulsed ultrasound (LIPUS) has led to several fascinating articles in which a variety of cell and tissue types were targeted, and the resultant physiologic responses and effects noted (reviewed in [136]). Recent studies have advocated targeting Piezo1 stimulation by LIPUS on macrophages [137] and endothelial cells [138]. More recently, in several clinically relevant experimental models, LIPUS stimulation has been applied to endothelial cells demonstrating promotion of angiogenesis [139,140]. Several recent studies note a reduction in tissue inflammation with the use of LIPUS. One potential mechanism for explaining this reductive inflammatory effect points to regulating macrophage polarization [137,141,142,143]. This therapeutic means may hold promise in chronic wounds where a paucity of M2 macrophages as well as impaired angiogenesis is pathophysiologically critical. 

As noted earlier, topical vacuum-assisted wound therapy can certainly be envisioned as a form of mechanotherapy improving angiogenesis and granulation tissue formation [96,113,144]. Under the assumption that Piezo1 is involved in this process, topically applied cyclic stress may augment the observed angiogenic results in static vacuum therapy. 

Skin (tissue) expansion, yet another clinically available form of mechanotherapy has long and widely been employed to induce additional skin and soft tissue for reconstructive surgical procedures. To the best of our knowledge, thus far, this modality has not been employed in the acute wound healing setting for additional mitogenic generation of a keratinocyte pool.

During the maturational phase of wound healing, it has long been recognized that excessive mechanical tension can play a significant role in the development of fibrosis. Correspondingly, the active mechanical offloading of the scar has demonstrated efficacy in both animal and clinical trials, resulting in significant improvement in scar formation. The offload of stretch exerted by underlying muscle tone has gained clinical attention [145,146], where locally injected botulinum toxin injection was reported to significantly improve scar quality. Mechanically, a commercially available device (Embrace, Neodyne Biosciences, Inc.) is a specialized dressing that adheres to the skin to offload the acute incisional site and improve its quality [6,21]. The mechanical compression of scars has long been advocated and employed for its beneficial effect on reducing scar volume [128]. Recently, it was shown that compression-induced apoptosis in periodontal fibroblasts could be reduced by blocking Piezo1 with the neurotoxin inhibitor GsMTx4 [147], possibly offering a novel pharmacological therapy. 

In addition to mechanical therapeutic approaches, biochemical interactions have similarly been employed in both agonist, as well as antagonist, strategies on Piezo channels [148]. The Piezo blocking agent, GsMTx4, has been intradermally administered to improve scar formation [112]. In an animal study, a 5 µM solution of GsMTx4 demonstrated efficacy in reducing scar volume by half. In an effort to minimize the risk of potential adverse events, the safe and efficacious application of such strategies will likely favor local application over systemic administration of agonists or antagonists. Thus far, and to the best of our knowledge, wound healing studies examining the administration of the Piezo1 agonists, Yoda1 or Yedi1 [149], or the antagonist GsMTx4, are lacking. A current search of ClinicalTrials.gov for clinical studies related to Piezo1 and wound healing have similarly revealed zero results [150].

The electrical stimulation of cutaneous wounds is an emerging and promising therapy for accelerating wound healing [4,5]. This electroceutical therapy has been applied to modulate cellular functions in macrophages [151], fibroblasts [152] and keratinocytes [153,154], with in vitro evidence for the involvement of Piezo1-mediating electroceutical therapy in endothelial cells as well as fibroblasts, leading to the proliferation and migration of these cell types [155]. 

In summary, there is mounting evidence supporting the integral role of mechanobiology in wound healing. Evolving strategies will most likely provide novel and additional approaches to accelerate or at least improve wound healing. Translating these effects may entail a wide variety of available and promising mechanically, electrically, magnetically or acoustically directed energy forms, as well as evolving physiologic, genetic, pharmacologic, and molecular agents. Numerous mechanoreceptors have now been established as promising modulators and effectors with the Piezo1 mechanoreceptor perhaps being the most well known. 

Problematic and impaired wound healing, scarring, challenging hemodynamic and inflammatory conditions, and pain afflict all biologic organisms. Future studies are strongly warranted. With our expanding understanding of the multidimensional influences involved in wound and regenerative healing, understanding and ultimately modulating the contributions of mechanosensors will likely open new doors and options for our patients and health care providers. Biophysical stimulation via Piezo1 may represent an attractive therapeutic means, the results of which will surely be explored in the coming years.

## Data Availability

No new data were created.
